# Low- and High-Volume of Intensive Endurance Training Significantly Improves Maximal Oxygen Uptake after 10-Weeks of Training in Healthy Men

**DOI:** 10.1371/journal.pone.0065382

**Published:** 2013-05-29

**Authors:** Arnt Erik Tjønna, Ingeborg Megaard Leinan, Anette Thoresen Bartnes, Bjørn M. Jenssen, Martin J. Gibala, Richard A. Winett, Ulrik Wisløff

**Affiliations:** 1 K.G. Jebsen Center for Exercise in Medicine at Department of Circulation and Medical Imaging, Trondheim, Norway; 2 Department of Biology, Norwegian University of Science and Technology, Trondheim, Norway; 3 Department of Kinesiology, McMaster University, Ontario, Canada; 4 Center for Research in Health Behaviour, Department of Psychology, Virginia Tech, Blacksburg, Virginia. United States of America; University of Bath, United Kingdom

## Abstract

Regular exercise training improves maximal oxygen uptake (VO_2max_), but the optimal intensity and volume necessary to obtain maximal benefit remains to be defined. A growing body of evidence suggests that exercise training with low-volume but high-intensity may be a time-efficient means to achieve health benefits. In the present study, we measured changes in VO_2max_ and traditional cardiovascular risk factors after a 10 wk. training protocol that involved three weekly high-intensity interval sessions. One group followed a protocol which consisted of 4×4 min at 90% of maximal heart rate (HR_max_) interspersed with 3 min active recovery at 70% HR_max_ (4-AIT), the other group performed a single bout protocol that consisted of 1×4 min at 90% HR_max_ (1-AIT). Twenty-six inactive but otherwise healthy overweight men (BMI: 25–30, age: 35–45 y) were randomized to either 1-AIT (n = 11) or 4-AIT (n = 13). After training, VO_2max_ increased by 10% (∼5.0 mL⋅kg^−1^⋅min^−1^) and 13% (∼6.5 mL⋅kg^−1^⋅min^−1^) after 1-AIT and 4-AIT, respectively (group difference, p = 0.08). Oxygen cost during running at a sub-maximal workload was reduced by 14% and 13% after 1-AIT and 4-AIT, respectively. Systolic blood pressure decreased by 7.1 and 2.6 mmHg after 1-AIT and 4-AIT respectively, while diastolic pressure decreased by 7.7 and 6.1 mmHg (group difference, p = 0.84). Both groups had a similar ∼5% decrease in fasting glucose. Body fat, total cholesterol, LDL-cholesterol, and ox-LDL cholesterol only were significantly reduced after 4-AIT. Our data suggest that a single bout of AIT performed three times per week may be a time-efficient strategy to improve VO_2max_ and reduce blood pressure and fasting glucose in previously inactive but otherwise healthy middle-aged individuals. The 1-AIT type of exercise training may be readily implemented as part of activities of daily living and could easily be translated into programs designed to improve public health.

**Trial Registration:**

ClinicalTrials.gov NCT00839579

## Introduction

The global epidemic of overweight [body mass index (BMI) between 25.0–29.9] and obesity (BMI≥30) has become a major health, social and economic burden. It is estimated that at least 400 million adults are obese, and approximately 1.6 billion adults are overweight [1]. Overweight and obesity are associated with increased mortality from cardiovascular and metabolic causes [2], [3], [4], whereas exercise training protects against premature cardiovascular mortality [5]. Current public health guidelines generally recommended that adults accumulate at least 150 min per week of moderate intensity exercise (50%–70% of HR_max_) or a minimum of 20 min of vigorous exercise (70% to 80% of HR_max_) at least three times per week [6]. These recommendations appear difficult to achieve for most people with one of the most common cited barriers being lack of time [7].

There is evidence to suggest that a lower volume of exercise may confer health benefits. For example, Lee et al. [8] showed that apparently healthy elderly men who exercised once or twice per week (so-called “weekend warriors”) had a lower risk of all-cause mortality compared with sedentary counterparts. Consistent with these findings, a 18-year follow-up study revealed that a single, vigorous weekly bout of physical activity was associated with prevention of cardiovascular death among men and woman without known cardiovascular disease at the beginning of follow-up [9]. These data suggest that it may be possible to reduce cardiovascular mortality with substantially less exercise than is generally recommended, provided it is performed in a vigorous manner [10].

Although both overweight and VO_2max_ are strong and independent prognostic markers of cardiovascular mortality, the link between VO_2max_ and mortality seems to be stronger [11]. Moreover, recent analyses have shown that while meeting physical activity recommendations marginally reduced all-cause mortality risk, being physically fit (as reflected by VO_2max_) was associated with a marked reduction in all-cause mortality risk even when physical activity was below recommendations [12], [13]. It has therefore been suggested that improving VO_2max_ is more important than losing weight or simply engaging in increasing amounts of lower to moderate intensity physical activity [12], [14]. These studies by different research groups call into question public health policies and programs largely revolving around accumulating a daily volume of lower to moderate physical activity.

There is evidence that exercise programs that involve relatively high intensity are more effective in improving VO_2max_, cardiac and endothelial function than isocaloric exercise programs of moderate intensities, in healthy individuals [15], [16], [17], patients with post-infarction heart failure [18], metabolic syndrome [19], coronary artery disease [20], and overweight and obese individuals [21]. We have demonstrated that an interval training program consisting of a 10-min warm-up followed by four, 4-min intervals at ∼90% of HR_max_ interspersed by 3 min of active recovery at ∼70% of HR_max_, performed 2–3 times per week for 8–16 weeks improved VO_2max_ by 16–46%. Endothelial function measured as flow mediated dilatation (FMD) also improved by 2–9% (absolute changes) in individuals with initially low FMD. These are relatively large adaptations over a short time period.

With regard to the practical translation of results from interval training studies into public health practice, a fundamental question remains: how abbreviated can the stimulus be, and still achieve a robust training effect measured as increased VO_2max_ and/or FMD and improvement in other cardiovascular disease (CVD) risk factors? One study found that after a several minute graded warm-up, simply training for several minutes at about 75% of VO_2max_ (∼85% of HR_max_) twice per week for 12 weeks was sufficient to increase the VO_2max_ of previously sedentary men and women by about 5 ml·kg^−1^·min^−1^ [22]. However, the brief high-intensity training intervention was only one aspect of a more comprehensive multi-component health behaviour program. It is not clear how effective such brief training can be when performed alone.

The present investigation was a small, proof-of-principle study designed to assess whether training using a brief single bout of high intensity exercise (1×4 min at 90% of HR_max_; 1-AIT) could improve VO_2max_ and elicit favourable health-related outcomes comparable to the standard 4×4 protocol (4-AIT) employed in our earlier studies. Therefore, the aim of this study was to determine the effects of two different interval training protocols, each performed 3 times per week for 10 weeks, on VO_2max_, FMD and other CVD risk factors in middle aged healthy men.

## Materials and Methods

The protocol for this trail and supporting CONSORT checklist are available as supporting information; see checklist S1 and protocol S1.

### Participants

**Figure 1:**
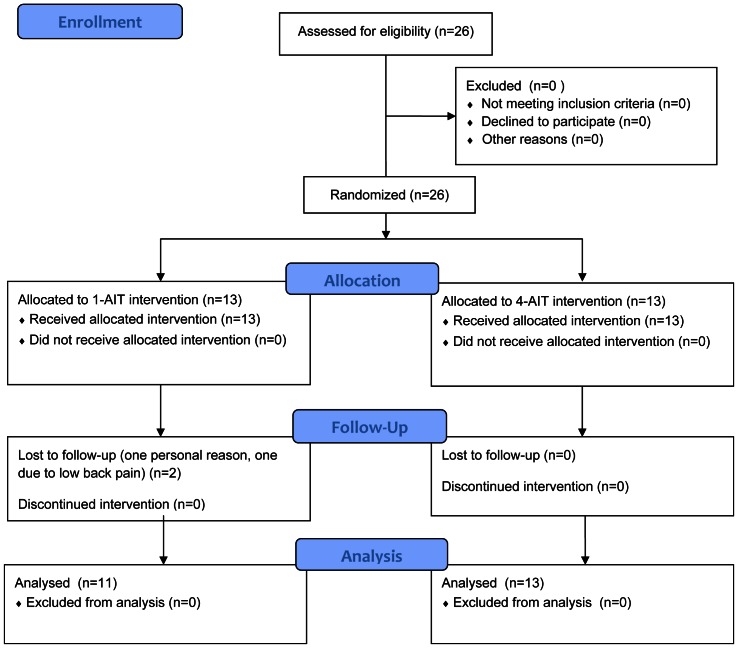
Flow chart of the study design.

Twenty-six inactive but otherwise healthy overweight men (BMI: 25–30, age: 35–45 years) were recruited for this investigation at St. Olav's hospital, Trondheim, Norway (study period: 05.01.2009–03.04.2009). The subjects had not exercised regularly for a period of at least two years prior to inclusion. Exclusion criteria were unstable angina, recent cardiac infarction (within 4 weeks), uncompensated heart failure, severe valvular illness, pulmonary disease, uncontrolled hypertension, kidney failure, orthopedic/neurological limitations, cardiomyopathy, and planned operations during the research period, reluctant to sign the consent from, drug or alcohol abuse or participants in a parallel study. All participants provided written, informed consent, and the regional committee for medical research ethics approved the protocol (21.11.2008). Participants were randomly assigned to either a single 4 min bout of exercise (1-AIT, n = 13) or 4×4 min bouts of exercise interspersed with 3 min recovery (4-AIT, n = 13), 3 times per week for 10 weeks (Figure 1). The randomization code was developed using a computer random number generator to select random permuted blocks. The unit of Applied Clinical Research at the university carried out all randomization procedures to secure complete blinded randomization. One participant in 1-AIT dropped out due to low back pain and one other moved to another part of the country and could not fulfill the training regime, which left 11 participants available for analysis in this group.

### Maximal oxygen uptake (VO_2max_), work economy and oxygen pulse

Before measurements of VO_2max_, participants were informed about the test, and instructed to exercise to their volitional maximal capacity. Familiarization procedures, warm-up, and the VO_2max_ ramp-procedure have been described in detail previously [20]. The mean of the three continuously highest 10 sec measurements was used to determine VO_2max_, while oxygen pulse was calculated as ((VO_2max_/HR_max_)×100). A leveling-off of oxygen uptake despite increased workload and a respiratory exchange ratio >1.05 were used as criteria for reaching the true VO_2max_, and this was achieved in all individuals in the present study. Work economy was defined as the oxygen cost of a 5-min walk at a 4 km·h^−1^ on a leveled treadmill. Oxygen uptake stabilized after 2–3 minutes at 4 km·h^−1^ and the average of the two last minutes were used in the data analysis. Post testing was performed between 3–5 days after last training session, and at the same time of the day as baseline test. The heart rate during the test and training sessions' was monitored by a Polar RS 400 (Polar Electro, Kempele Finland) and the highest HR value during the test were defined as HR_max_.

### Training protocols

Both groups trained by walking/jogging/running on an inclined treadmill 3/wk. Following a 10 min warm-up at an intensity that elicited 70% of HR_max_, subjects performed either 1-AIT or 4-AIT at 90% of HR_max_. The work bouts in the 4-AIT group were interspersed by 3 min of active recovery at 70% HR_max_. Both groups performed a 5 min cool-down. Total exercise time per session was therefore 19 and 40 min, respectively for the 1-AIT and 4-AIT groups.

### Endothelial function and blood pressure

FMD was measured in the brachial artery using high-resolution vascular ultrasound (14 MHz echo Doppler probe, Vivid 7 System, GE Vingmed Ultrasound, Horten, Norway) according to the current guidelines [23]. The procedures for measuring FMD and blood pressure were recently published by our group [21]. Shear rate was calculated as blood flow velocity/diameter (cm⋅s^−1^·cm^−1^) as previously published [24]. Since no group differences were found for shear rate, data are not presented. All ultrasound images were analyzed in random order, using EchoPACtm (GE Vingmed Ultrasound AS, Horten, Norway) by an investigator blinded to the subjects' group allocation.

### Blood analyses

Blood samples were obtained in the fasted state (≥8 h) and plasma triglycerides, high-density lipoprotein cholesterol, total cholesterol, hemoglobin, high-sensitive C-reactive protein, glycosylated hemoglobin (HbA1c), glucose and insulin C-peptide were analyzed using standard local procedures at St. Olav's University Hospital, Trondheim, Norway. Non-fasting glucose was analyzed by Hexokinase/G-G-PDH methodology reagent kit 3L82-20/3L82-40 Glucose, high-density lipoprotein (HDL) cholesterol by the Accelerator selective detergent methodology reagent kit 3K33-20 Ultra HDL, triglycerides by Glycerol Phosphate Oxidase methodology reagent kit 7D74 Triglyceride, alanine aminotransferase (ALAT) by NADH (with P-5′-P) methodology reagent kit 8D36-30 Alanine aminotransferase activated, aspartate aminotransferase (ASAT) by NADH (with P-5′-P) methodology reagent kit 8D37-30 Aspartate aminotransferase activated, and C-reactive protein (CRP) was analysed by the Areoset CRP Vario kit (all analyses from Abbott Diagnostics, Illinois, US). Oxidized LDL was measured in plasma with the Mercodia oxidized-LDL ELISA (Mercodia, Uppsala, Sweden).

### Body Composition

BMI was calculated and dual-energy X-ray absorptiometry (Dxa, Hologic Discovery A, WA, USA) scanning was used to determine body composition.

### Statistical analyses

The primary outcome variable was VO_2max_. Prior experience suggests a standard deviation (SD) of about 2.0–3.0 ml/kg/min [18]. According to sample size tables for clinical studies, we needed 10 subjects in each group (we included 13 in case of drop out) With a standardized within-group difference of 1.0 differences may be detected using a paired t-test with 80% power, at a significance level of 5% [25]. Clinically, this corresponds to a detectable difference for VO_2max_ of 3 ml/kg/min. Wilcoxon's non-parametric procedures were used to analyze effect of intervention if the assumption of normality and homogeneity of variance was in doubt. For group differences between groups in the training-induced changes, mixed linear model analyses with group and time interaction were performed. Reported p-values are two-sided, and refer to mixed model analyses and values ≤0.05 are considered as statistically significant. SPSS® 16.0 (SPSS Inc. Chicago, IL.) was employed for all analyses.

## Results

### Baseline characteristics

Baseline characteristics were similar in the two groups. However, despite randomization, there was a tendency for higher body mass in 1-AIT when compared to 4-AIT (Table 1).

**Table 1:** Subjects baseline values (mean, SD), and mean within group change (95% CI) post exercise intervention.

	BL 1-AIT	Post 1-AIT	BL 4-AIT	Post 4-AIT	P-value
**Age** (years)	41.8±3.6		42.2±2.4		
**BODY COMPOSITION**					
Height, cm	185±6.9		179±8.4		
Weight, kg	95.1±5.0	−1.8 (−3.3, −0.3)	85.9±8.9	−2.1 (−3.2, −1.1)	0.19
BMI, kg/m^2^	27.8±1.8	−0.4 (−0.7, −0.1)	27.0±2.1	−0.7 (−0.9, −0.4)	0.56
Fat percent, %	25.7±3.6	−0.6 (−1.2, 0.1)	23.0±2.9	−0.8 (−1.4, −0.2)	0.89
Fat weight trunk, kg	13.2±1.7	−0.5 (−1.3, 0.3)	10.6±2.1	−0.9 (−1.6, −0.3)	0.62
Fat weight total, kg	24.5±2.9	−0.7 (−1.7, 0.3)	19.9±3.5	−1.1 (−1.9, −0.3)	0.98
**MAXIMAL OXYGEN UPTAKE**					
VO_2max,_, mL · kg^−1^ · min^−1^	39.5±5.1	4.5 (2.4, 6.5)	44.8±5.1	7.0 (5.1, 8.8)	0.08
**BLOOD PRESSURE/FMD**					
Flow mediated dilatation, %	4.85±3.7	−0.5 (−1.9, 1.0)	5.62±4.17	−0.8 (−2.2, 0.5)	0.75
Systolic Blood Pressure, mmHg	142.4±17.6	−6.2 (−11.4, −0.9)	136.3±11.7	−3.2 (−8.1, 1.7)	0.84
Diastolic Blood Pressure, mmHg	91.9±8.7	−7.7 (−15.1, −0.3)	88.4±7.1	−6.3 (−13.2, 0.5)	0.79
Mean arterial blood pressure, mmHg	108.7±11.2	−7.2 (−13.0, −1.4)	104.4±7.8	−5.1 (−10.5, 0.3)	0.99
**BLOOD VARIABLES**					
Triglyceride, mmol · L^−1^	1.21±0.47	0.12 (−0.16, 0.39)	1.22±0.60	0.07 (−0.19, 0.33)	0.77
HDL-cholesterol, mmol • L^−1^	1.31±0.27	−0.06 (−0.15, 0.04)	1.47±0.44	−0.11 (−0.19, −0.02)	0.41
Fasting Glucose, mmol • L^−1^	5.13±0.41	−0.35 (−0.61, −0.08)	5.28±0.34	−0.25 (−0.49, −0.01)	0.80
Hemoglobin, g • dL^−1^	15.3±0.69	−0.07 (−0.36, 0.23)	15.5±0.83	−0.20 (−0.46, 0.07)	0.48
Cholesterol, mmol • L^−1^	6.56±1.36	−0.31 (−0.92, 0.31)	6.36±0.95	−0.54 (−1.10, 0.03)	0.71
LDL-cholesterol, mmol • L^−1^	4.70±1.30	−0.29 (−0.85, 0.27)	4.34±0.86	−0.44 (−0.96, 0.08)	0.96
ox-LDL cholesterol, U/L	101.2±24.2	−6.36 (−13.11, 0.39)	98.3±15.5	−14.77 (−20.98, −8.56)	0.07
HbA1c, %	5.44±0.19	0.09 (0.02, 0.17)	5.51±0.17	0.13 (0.07, 0.19)	0.36
C-peptide, nmol • L^−1^	0.72±0.38	−0.03 (−0.20, 0.14)	0.58±0.30	−0.01 (−0.18, 0.15)	0.67
CRP, mg • L^−1^	2.26±1.70	−0.54 (−0.97, −0.10)	1.64±1.74	−0.98 (−1.38, −0.58)	0.36

Baseline data are presented as mean ± SD. Post values are mean within group change (95% CI) after exercise intervention. P-values are from mixed model analyses after adjusting for baseline values. BL; baseline. HbA1c; glycosylated hemoglobin. CRP: c - reactive protein, FMD; flow mediated dilatation (1-AIT, n = 11; 4-AIT, n = 13).

### Clinical follow up

#### Maximal oxygen uptake (VO_2max_) and work economy

**Figure 2:**
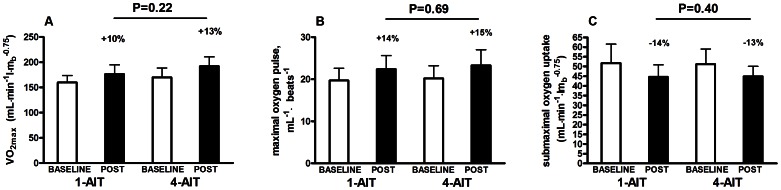
Maximal oxygen uptake (VO_2max_) (A). Maximal oxygen pulse (B). Work economy presented as submaximal oxygen uptake (C). Data represents mean (SD).

1-AIT and 4-AIT increased *V*O_2max_ by 10% (17.2 (9.5, 24.9)) and 13% [22.9 (16.1, 29.7) with no group differences (p = 0.23), Figure 2A]. Similarly, stroke volume indicated by peak oxygen pulse increased by 14% (2.6 (1.7, 3.6)) after 1-AIT and 15% [3.1 (2.2, 4.0) after 4-AIT (group differences, p = 0.7), Figure 2B]. Work economy improved by 14% (−6.9 (−10.0, −3.9)) and 13% [−6.4 (−9.2, −3.6) after 1-AIT and 4-AIT, respectively (group differences, p = 0.4), Figure 2C].

#### Flow Mediated Dilatation of the brachial artery (FMD)

There were no group differences, and no effect of training, on shear rate or basal diameter of the brachial artery, or FMD (Table 1).

#### Anthropometry

1-AIT and 4-AIT decreased BMI and body weight by 1% (−0.4 (−0.7, −0.1)) and 2% [−0.7 (−0.9, −0.4), respectively (Table 1), group differences, p = 0.56]. Fat percent decreased by 2% (−0.6 (−1.2, 0.1)) in 1-AIT and 3% (−0.8 (−1.4, −0.2)) in 4-AIT [Table 1, (group differences, p = 0.89)]. Fat weight trunk decreased 5.5% (−0.5 (−1.3, 0.3)) and 7.9% (−0.9 (−1.6, −0.3)) after 1-AIT and 4-AIT respectively, (Table 1, group differences, p = 0.62), while total fat weight decreased by 3.2% (−0.7 (−1.7, 0.3)) after 1-AIT and 5.2% (−1.1 (−1.9, −0.3)) in 4-AIT (Table 1, group differences, p = 0.98).

#### Blood pressure

Both 1-AIT and 4-AIT decreased mean arterial blood pressures, by ∼6 mmHg [−13.0, 0.3) (Table 1) group differences, p = 0.99]. Systolic blood pressure decreased with 6.2 (−11.4, −0.9) and 3.2 (−8.1, 1.7) (group differences, p = 0.84) mmHg in 1-AIT and 4-AIT respectively, whereas diastolic blood pressure decreased with 7.7 (−15.1, −0.3) and 6.3 (−13.2, 0.5) (group differences, p = 0.79) mmHg in 1-AIT and 4-AIT respectively (Table 1).

#### Blood analysis

1-AIT and 4-AIT reduced fasting glucose by 6% [−0.35 (−0.61, −0.08)] and 5% (−0.25, (−0.49, −0.01)), respectively [Table 1, (group differences: p = 0.80)], while cholesterol, LDL-cholesterol, and ox-LDL cholesterol only was reduced after 4-AIT [Table 1, (group differences: p = 0.71, 0.96 and 0.07 respectively)].

## Discussion

The main finding from the present study was that a single bout of exercise that consisted of 1×4 min at 90% HR_max_ (1-AIT) increased VO_2max_ to a similar extent as 4×4 min at 90% HR_max_ (4-AIT), when both protocols were performed 3×/wk for 10 wk in healthy overweight middle-aged men. Both interventions also induced similar improvements in submaximal work economy, blood pressure and fasting plasma glucose, but 4-AIT was more effective in reducing blood cholesterol and body fat.

### VO_2max_ oxygen pulse and work economy

The present study demonstrates that a relatively intense stimulus administered only once and for a relatively short duration can substantially improve VO_2max_ and work economy. A single bout of 4-minute interval training three times per week will not solve all lifestyle-related problems for people already obese or overweight, and it is not the only solution for inactive persons with a BMI below 25. However, brief interval training can have a central role in public health and lifestyle medicine initiatives, in addition to changes in nutrition and other, less intense physical activity. This is because of all established risk factors, low VO_2max_ seems to be the strongest predictor of mortality [4] and improvements in VO_2max_ are associated with reduced mortality risk [4], [11]. Epidemiologic studies have shown that each 1-MET (∼3.5 mL·kg^−1^·min^−1^) increase in exercise capacity confers an 8% to 17% reduction in cardiovascular and all-cause mortality [4], [26], [27]. Increased VO_2max_ was associated with increased maximal peak O_2_ pulse after both exercise programs, indicating increased maximal stroke volume was a central mechanism for improved VO_2max_ [28]. These data are consistent with previous studies applying the 4-AIT protocol [15]. However, this is the first study to show such improvements also may occur with substantially less training volume than previously thought. Improved work economy was in line with previous studies from our group [18], [19] but the surprising observation was that 1-AIT improved it to a similar extent as 4-AIT. The cause of intra-individual variations in gross oxygen cost of activity at a standard workload are not very well understood, but it seems likely that anatomical traits, mechanical skill, neuromuscular skill and storage of elastic energy are important [29].

### Flow mediated dilatation (FMD)

Aerobic exercise training is known to improve FMD when it is reduced due to age, metabolic syndrome, heart failure etc. [18], [19]. FMD observed in the present study was within the normal range for this age group [30]. Additionally, no effect of exercise training on FMD in healthy individuals is consistent with previous studies indicating relatively little or no effect when FMD is well preserved at baseline [31], [32].

### Blood pressure

The observed blood pressure lowering also is consistent with previous studies [19], [33].

According to a large meta-analysis, a blood pressure lowering of the magnitude observed in our study would in the long-term, be associated with a large decrease of premature deaths resulting from stroke and ischemic heart disease [34].

### Blood analysis

The magnitude of reduced plasma glucose, both after 1-AIT and 4-AIT are similar to findings by Babraj et al. showing that extremely short durations of high intensity interval training reduced plasma glucose values [35]. Furthermore the reduction in plasma oxidized LDL and cholesterol levels together with decreased trunk fat may indicate improvement in the fat metabolism as previously demonstrated [19].

### Anthropometry

Reduced body mass and improved body composition after 4-AIT is consistent with previous studies [19], [21], [36], [37], [38], [39]. Surprisingly, the 1-AIT induced similar though smaller changes in percent body fat and trunk fat. This is a compelling finding because it suggests that a brief, but higher intensity stimulus may be sufficient over time for inducing changes, albeit modest ones, in body composition.

### Practical implication and safety

The 1-AIT type of exercise training may be readily implemented in activities of daily living at least for relatively untrained individuals. The 1-AIT corresponds to a 4-minute quick walk up-hill at ∼8% to 10% grade (to or from work/school) or rapidly walking up 6–10 flights of stairs 3/wk. Rather than heart rate monitors, public health programs can emphasize higher levels of perceived exertion (using standard scales) during the 4-minute interval. A rating of about 16 on the Borg-scale would correspond to about 90% of maximal heart rate [18].

Our previous studies showed that most individuals can engage in this type of exercise training as it has been performed without any adverse events in obese and overweight individuals [21], in persons with the metabolic syndrome [19], in patients with coronary artery disease [20], patients with intermittent claudication [40], or after coronary artery bypass surgery [41]. Additionally a recently safety study concluded that high intensity exercise should be considered among patients with coronary heart disease [42].

### Limitations of the study

The present study is an initial efficacy study with a small group of healthy participants and potentially under-powered to detect small though possibly clinically meaningful differences between groups. However the results suggested that short duration, but intense training can yield favourable, potentially risk reduction benefits. Longer-term systematic replication studies with diverse participants are needed to more fully assess impacts on risk factors and at the molecular level. If such studies showed consistent positive outcomes, then translational research studies would be needed to assess the impact of such brief, but intense training within less supervised settings and as part of lifestyle interventions as suggested previously.

## Conclusion

Our study demonstrated that slightly overweight and healthy individuals only required brief, duration bouts of exercise with good effort three times a week, to produce large increases in VO_2max_ and work economy and reduce blood pressure and fasting glucose levels. Additional studies to examine both adaptations at the molecular level and feasibility for public health appear warranted.

## Supporting Information

Checklist S1 CONSORT Checklist.
(DOC)

Protocol S1 Trial Protocol.
(PDF)
