# Supplementary material for: Low- and High-Volume of Intensive Endurance Training Significantly Improves Maximal Oxygen Uptake after 10-Weeks of Training in Healthy Men
Source: PLoS One. 2013 May 29;8(5):e65382. doi: 10.1371/journal.pone.0065382 (PMC3667025; doi:10.1371/journal.pone.0065382)
Supplement: Protocol S1 — Trial Protocol. (PDF) [file pone.0065382.s002.pdf]

Protocol S1

How little pain for cardiac gain?

BACKGROUND

The global epidemic of overweight and obesity has become a major health, social and economical burden with at least 400 million adults being obese defined as having a body mass index (BMI) ≥30, and approximately 1.6 billion adults (age 15+) being overweight; with a BMI between 25 and 29.9 [1]. Both overweight and obesity appear to be associated with low aerobic capacity and impaired endothelial function [2], both being strong and independent risk factors of mortality from cardiovascular and metabolic diseases [3-5]

Exercise training is proved to protect against premature cardiovascular mortality [6, 7]. Additionally there are evidence that relatively high exercise intensity may be an important factor for improving aerobic capacity and endothelial function in patients with post-infarction heart failure [8], metabolic syndrome [9], coronary artery disease [10], as well as in overweight and obese individuals [11].

Although both overweight and aerobic capacity are strong and independent prognostics markers of cardiovascular mortality, the link between aerobic capacity and mortality seems to be stronger [12], and it has therefore been suggested that improving aerobic capacity is more important than loosing weight per se [13].

Current guidelines recommended at least 30 min of daily exercise of moderate intensity, or 20 min of vigorous exercise at least three times per week, but these recommendations appear to be hard to fulfill for most people [14]. Interestingly, Lee et al. [15] showed that apparently healthy elderly men who exercised one to two times per week (so-called weekend warriors), had a lower risk of all-cause mortality compared with sedentary, apparently healthy men. In line with this, Wisløff et al [16] showed a significant prevention of cardiovascular death among men and woman without known cardiovascular disease by a single weekly bout of exercise training. Therefore, substantially less exercise than currently recommended [17] may be

sufficient to reduce cardiovascular mortality [18]. A central question has therefore been `how little can we get away with`, and implicitly, achieve protection against premature cardiovascular disease.

AIM OF STUDY

The aim of this study will be to achieve more knowledge about the amounts of high-intensity exercise needed to improve aerobic capacity. To catch up with this knowledge, the effect of the well known high-intensity interval exercise protocol 4 x 4 min of ~90% of VO_{2max}, three times a week, will be challenged with a high-intensity interval exercise protocol containing 1x 4 min of ~90% of VO_{2max}, three times a week. Previous studies have demonstrated no difference with regard to strength and muscle mass gains between 1- and 3-set strength training in upper-body muscles in untrained men [19]. Therefore, we hypothesize that 1 x 4 minutes will give the same improvement in VO_{2max} as the traditional 4 x 4 minutes regimen.

METHODS

Subjects

With an expected change in mean VO_{2max} of 8ml/min^{-1}/kg^{-1} (SD: 5), and a p-value on 0.05, ten individuals in each group will give us a statistical power of 92%. Twenty male subjects (age 35-45 years) will therefore be randomized into two different training regimes.

The subjects will go through a standard medical examination before inclusion.
Inclusion criteria will be healthy males with BMI: 25-30. Exclusion criteria will be unstable angina pectoris, myocardial infarction within the last 12 months, decompensated heart failure, cardiomyopathy, severe valvular heart disease, considerable pulmonary disease, uncontrolled hypertension, kidney failure, orthopedic and/or neurological limitations to exercise, surgery during the intervention period, drug or alcohol abuse, or participation in another research study. A compliance with the training program of 70% will also be set as a criterion for completing the study.

Protocol S1

Study design

The subjects will be randomized to either high intensity aerobic interval training, 4 x 4 min (n=10) or high intensity aerobic interval training, 1x 4 min (n=10). Both groups will exercise 3 times per week over a 12-week period; all of them supervised by the study investigators in a research laboratory at MTFS, NTNU.

Aerobic training

Exercise training in both groups will be treadmill walking or running. The training protocol will start with a 10 min warm-up period at ~60% of maximal heart rate (HR_{max}) for both groups. Following 4 x 4-min intervals at 85-95 % of HR_{max} with 3 min active breaks in between the intervals, at ~60% of HR_{max} for group one. The exercise session will be terminated by a 5 min cool-down period. The second group will, after the warm-up period, perform a 1 x 4-min interval at 85-95 % of HR_{max} followed by a 5 min cool-down. The subjects will be instructed to control the intensity by monitoring heart rate (HR) and thereby adjusting the speed and/or inclination of the treadmill to correspond to the preferred exercise intensity. For each session, HR, speed and inclination will be recorded. Lactate measurements will be done every other week, as intensity control.

Maximal oxygen uptake

VO_{2max} will be measured during uphill treadmill walking or running (Woodway PPS 55 Med, Munich, Germany), using ergospirometry (Jaeger, Oxycon pro, Hoechberg, Germany), as described earlier [10]. A warm-up period for 10 min (~60% of HR_{max}) will precede the test. A leveling off of oxygen uptake (VO_2) despite increased work load and respiratory exchange ratio ≥1.05 will be used as criteria for VO_{2max}. HR will be measured continuously during the test (Polar, Polar Electro, Kempele, Finland), to define HR_{max}.

Protocol S1

Biochemistry of muscle biopsies - peripheral O_2 use

Muscle biopsies will be obtained from *m.vastus lateralis* using a sterile 5 mm diameter biopsy needle [20] (Bergstrøm, Stille, Stockholm, Sweden) under local anaesthesia (2% Lidocaine). A 5- to 10 mm incision will be made; the Bergström needle introduced into the muscle tissue, and three to four cuts will be made. If present, superficial blood will be quickly removed, and the biopsy will be frozen in liquid nitrogen and stored at -80° C for later analysis for PCG-1α (Western Blot).

For measuring Ca^{2+} re-uptake into sarcoplasmic reticulum, Ca^{2+} (50 μmol/L) will be added to skinned muscle fibers from the vastus lateralis muscle to induce a rapid increase in [Ca^{2+}], and kinetics of the subsequent decline in [Ca^{2+}], will be analyzed with Fura-2 on an epi-fluorescence microscope (Diaphot-TMD, Nikon, Tokyo, Japan) to assess maximum SR Ca^{2+} ATPase (SERCA)-1 and -2 transport capacity.

DEXA

BMI will be calculated and dual-energy X-ray absorptiometry (Dexa, Hologic Discovery A, WA, USA) scanning will be used to determine body composition.

Blood pressure and blood analysis

Blood pressure will be measured while the patient is sitting down and have been resting for at least five minutes in a quiet room. It will be measured by a trained physiologist, with a handheld sphygmomanometer (Tycos, 5098-02CB, USA). Blood pressure will be measured at the same time of the day for each individual at pre- and post test. The first reading will be discarded and the mean of the next three consecutive readings with a coefficient of variation below 15% will be used in the study, with additional readings if required.

All blood analyses will be performed using standard local procedures.

Oxidized-LDL and adiponectin will be measured in plasma using specific ELISA kits (Mercodia, Uppsala, Sweden), total nitrite (NO_2-) concentration will be quantified using a commercially available assay for nitric oxide (NO)-detection (R&D systems, Inc., Minneapolis, MN, USA). To estimate β-cell function (%B) and overall insulin sensitivity (%S), the homeostasis assessment model (HOMA) will be used. This computer model gives a value for insulin sensitivity expressed as HOMA2-%S (where 100% is normal), which is simply the reciprocal of HOMA2-IR [21].

Protocol S1

Endothelial function

Endothelium- dependent and –independent dilatation will be studied according to the method originally described by Celermajer et al (Celermajer, Sorensen et al. 1992). Endothelial function of the artery will be measured as flow-mediated dilatation (FMD) using high- resolution vascular ultrasound (14 MHz ultrasound- Doppler probe, Vivid 7 system; GE Vingmed Ultrasound AS, Horten, Norway) according to current guidelines (Raitakari and Celermajer 2000; Corretti, Anderson et al. 2002). Endothelium- independent dilatation will be measured by administrating µg 500 glycerol trinitrate (GTN) sublingually. All ultrasound images will be analyzed in random order using EchoPACtm (GE Vingmed Ultrasound AS) by an investigator blinded to the group allocation of the subjects. Diameters will be measured from intima to intima using calipers with 0, 1 - mm resolution. The mean of three diameter measurements and flow measurements will be used in the calculation of FMD, GTN and flow- responses.

Protocol S1

REFERENCES

1. World Health Organization. *Obesity and overweight - What are overweight and obesity?* Media centre of WHO [online] . 2006. Fact sheet N°311. Accessible at: http://www.who.int/mediacentre/factsheets/fs311/en/ (Downloaded 14.10.08)

2. Kopelman PG. *Obesity as a medical problem*. Nature. 2000. **404**. 635-643

3. Deanfield JE, Halcox JP, Rabelink TJ. *Endothelial function and dysfunction: testing and clinical relevance*. Circulation. 2007. **115**. 1285-1295

4. Kvanagh T, Mertens DJ, Hamm LF, et al. *Prediction of long-term prognosis in 12 169 men referred for cardiac rehabilitation*. Circulation. 2002. **106**. 666-671

5. Myers J, Prakash M, Froelicher V, Do D, Partington S, Atwood JE. *Exercise capacity and mortality among men referred for exercise testing*. N. Engl. J. Med. 2002. **346**. 793-801

6. US Department of Health and Human Services. *Physical activity and health. A report of the surgeon General.* Atlanta, GA US Department of Health and Human Services, Centers for Disease Control and Prevention, National Center for Chronic Disease Prevention and Health Promotion; 1996.

7. Barengo NC, Hu G, Lakka TA, Pekkarinen H, Nissinen A, Tuomilehto J. et al. *Low Physical activity as a predictor for total and cardiovascular disease mortality in middle-aged men and woman in Finland.* Eur Heart J 2004; **24** 2204-2411

8. Wisløff U, Støylen A, Loennchen JP, Bruvold M, Rognno Ø, Haram PM. *Superior Cardiovascular Effect of Aerobic Interval Training Versus Moderate Continuous Training in Heart Failure Patients. A Randomized Study.* Circulation. 2007.**115.** 3086-3094.

9. Tjønna AE, Lee SJ, Rognmo Ø, Stølen TO, Bye A, Haram PM et al. An *Aerobic Interval Training Versus Continuous Moderate Exercise as a Treatment for the*

Metabolic Syndrome. A Pilot Study. Circulation. 2008. **118**. 346-354

10. Rognmo O, Hetland E, Helgerud J, Hof J, Slørdahl SA, *High intensity aerobic interval exercise is superior to moderate intensity exercise for increasing aerobic capacity in patients with coronary artery disease.* Eur. J. Cardiovasc. Prev. Rehabil. 2004. **11 (3)**. 216-222

11.. Schjerve IE, Tyldum GA, Tjønna AE, Stølen T, Loennechen JP, Hansen HEM et al. *Both aerobic endurance and strength training programs improve cardiovascular health in obese adults.* Clinical Science. 2008. **115 (9)**. 283-93

12. Blair SN and Brodney S. *Effects of physical inactivity and obesity on morbidity and mortality; current evidence and research issues.* Med. Sci. Sports Exerc. 1999. **31**. S646-S662

13. Gaesser GA. *Thinness and weight loss: beneficial or detrimental to longevity?* Med. Sci. Sports Exerc. 1999. **31**. 1118-1128

14. Trost SG, Oweb N, Bauman AE, Sallis JF, Brown W. *Correlates of adults` participation in physical activity: review and update.* Med Sci. Sports. Exerc. 2002. **34**. 1996-2001

15. Lee IM, Sesso HD, Oguma Y and Paffenbarger RS Jr. *Relative intensity of physical activity and risk of coronary heart disease.* Circulation. 2003. **107**. 1110-1116

16. Wisløff U, Nilsen TIL, Drøyvold WB, Mørkved S, Slørdahl SA, Vatten LJ. *A single weekly bout of exercise may reduce cardiovascular mortality: how little pain for cardiac gain? `The HUNT study, Norway`.* Eur. Jour. Of Card. Prev. and Rehab. 2006. **13**. 798-804

17. Fletcher GF, Balady GJ, Amsterdam EA et al. *Exercise standards for testing and training: a statement for healthcare professionals from the American Heart Association.* Circulation. 2001. **104**. 1694-1740

18. Opie LH. *The Heart, Physiology from cell to circulation.* Third Edition. Lippincott Williams and Wilkins. Philadelphia. 1998. 447-454

19. Rønnestad BR, Egeland W, Kvamme NH, Refsnes PE, Kadi F, Raastad T. *Dissimilar Effects of One- and Three-Set Strength Training on Strength and Muscle Mass Gains in Upper and Lower Body in Untrained Subjects.* The Journal of Strength and Conditioning Research. 2007. **21**. 157–163

20. Bergstrom, J. () *Percutaneous needle biopsy of skeletal muscle in physiological and clinical research*. Scand. J. Clin. Lab. Invest. 1975. **35**. 609–616

21. Wallace T. M., Levy J. C., Matthews D. R. *Use and abuse of HOMA modeling. Diabetes care.* 2004. **27**. 1487-1495.
